# 2-(2,5-Dichloro­benzene­sulfonamido)-3-methyl­butanoic acid

**DOI:** 10.1107/S1600536810041620

**Published:** 2010-10-23

**Authors:** Islam Ullah Khan, Peter John, Haffsah Iqbal, Shahzad Sharif, Edward R. T. Tiekink

**Affiliations:** a Materials Chemistry Laboratory, Department of Chemistry, Government College, University, Lahore 54000, Pakistan; b Department of Chemistry, University of Malaya, 50603 Kuala Lumpur, Malaysia

## Abstract

The structure of the title compound, C_11_H_13_Cl_2_NO_4_S, shows one sulfonamide-O atom to lie almost in the plane of the benzene ring [C—C—S—O = −178.7 (2) °] and the other to one side [C—C—S—O = −49.4 (3)°]. Lying to the other side is the amine residue, which occupies a position almost perpendicular to the plane [C—S—N—C = 70.2 (2)°]; the carb­oxy­lic acid group is orientated to lie over the benzene ring. In the crystal, the appearance of an 11-membered {⋯OH⋯OCOH⋯OC_2_NH} synthon, which features the hy­droxy group forming both donor (to a carbonyl-O) and acceptor (from the amine-H) inter­actions, leads to the formation of a supra­molecular chain along the *a* axis. Chains are connected in the crystal structure by C—H⋯O contacts.

## Related literature

For background to the pharmacological uses of sulfonamides, see: Korolkovas (1988 ▶); Mandell & Sande (1992 ▶). For related structures, see: Sharif *et al.* (2010 ▶); Khan *et al.* (2010 ▶).
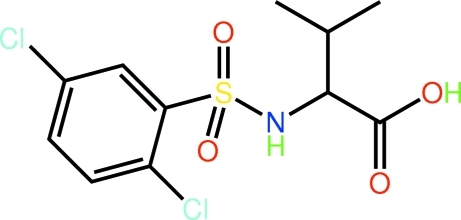


## Experimental

### 

#### Crystal data

C_11_H_13_Cl_2_NO_4_S
*M* *_r_* = 326.18
Orthorhombic, 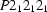

*a* = 5.4584 (2) Å
*b* = 14.0623 (6) Å
*c* = 19.4545 (8) Å
*V* = 1493.28 (10) Å^3^
*Z* = 4
Mo *K*α radiation
μ = 0.58 mm^−1^
*T* = 293 K
0.19 × 0.13 × 0.07 mm

#### Data collection

Bruker APEXII CCD diffractometer
Absorption correction: multi-scan (*SADABS*; Sheldrick, 1996 ▶) *T*_min_ = 0.805, *T*_max_ = 0.921
14542 measured reflections
3405 independent reflections
2876 reflections with *I* > 2σ(*I*)
*R*_int_ = 0.040

#### Refinement

*R*[*F* ^2^ > 2σ(*F* ^2^)] = 0.041
*wR*(*F* ^2^) = 0.127
*S* = 1.00
3405 reflections
180 parameters
2 restraints
H atoms treated by a mixture of independent and constrained refinement
Δρ_max_ = 0.61 e Å^−3^
Δρ_min_ = −0.51 e Å^−3^
Absolute structure: Flack (1983 ▶), 1415 Friedel pairs
Flack parameter: 0.09 (8)

### 

Data collection: *APEX2* (Bruker, 2007 ▶); cell refinement: *SAINT* (Bruker, 2007 ▶); data reduction: *SAINT*; program(s) used to solve structure: *SHELXS97* (Sheldrick, 2008 ▶); program(s) used to refine structure: *SHELXL97* (Sheldrick, 2008 ▶); molecular graphics: *ORTEP-3* (Farrugia, 1997 ▶) and *DIAMOND* (Brandenburg, 2006 ▶); software used to prepare material for publication: *publCIF* (Westrip, 2010 ▶).

## Supplementary Material

Crystal structure: contains datablocks global, I. DOI: 10.1107/S1600536810041620/hg2730sup1.cif

Structure factors: contains datablocks I. DOI: 10.1107/S1600536810041620/hg2730Isup2.hkl

Additional supplementary materials:  crystallographic information; 3D view; checkCIF report

## Figures and Tables

**Table 1:** Hydrogen-bond geometry (Å, °)

*D*—H⋯*A*	*D*—H	H⋯*A*	*D*⋯*A*	*D*—H⋯*A*
O3—H3o⋯O4^i^	0.82 (3)	1.86 (2)	2.674 (3)	171 (3)
N1—H1n⋯O3^ii^	0.85 (2)	2.32 (2)	3.161 (3)	167 (3)
C7—H7⋯O1^iii^	0.98	2.42	3.341 (3)	157
C4—H4⋯O2^iv^	0.93	2.41	3.223 (5)	146

Symmetry codes: (i) 
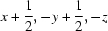
; (ii) 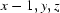; (iii) 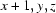; (iv) 
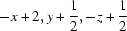
.

**Fig. 1.**
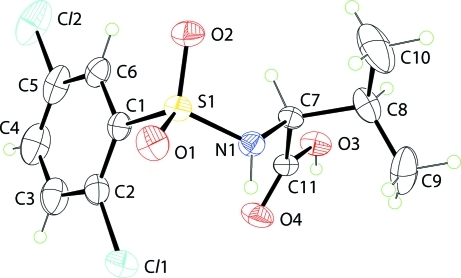
The molecular structure of (I) showing the atom-labelling scheme and displacement ellipsoids at the 35% probability level.

**Fig. 2.**
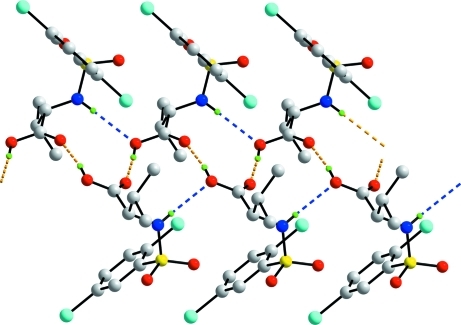
A view of the linear supramolecular chain along the a axis in (I). The O–H···O and N–H···O hydrogen bonds are shown as orange and blue dashed lines, respectively.

**Fig. 3.**
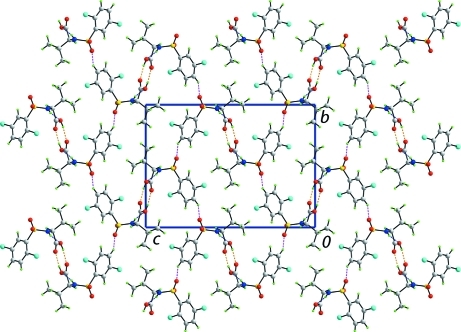
View in projection down the a axis of the unit-cell contents for (I). The O–H···O, N–H···O and C—H···O contacts are shown as orange, blue and pink dashed lines, respectively.

## supplementary crystallographic information

### Comment

The crystal structure of the title compound, (I), was determined in connection with on-going structural studies of sulfonamides (Sharif *et al.*, 2010; Khan *et al.*, 2010), of interest owing to their biological properties (Korolkovas, 1988; Mandell & Sande, 1992).

With reference to the benzene ring in (I), Fig. 1, the O2 atom lies in the plane [the O2—S1—C1—C2 torsion angle = -178.7 (2) °] but the O1 atom lies out of the plane [O1—S1—C1—C2 = -49.4 (3) °]. The amine group lies to the opposite side of the plane to the O1 atom and occupies a position almost perpendicular to it [C1—S1—N1—C7 = 70.2 (2) °]. Within the amine residue, the carboxylic acid group is co-planar with the amine-N1 [N1—C7—C11—O4 = -1.6 (4) °], and is folded to be orientated over the benzene ring with the carbonyl-O4 atom closest to it.

In the crystal packing, the hydroxyl-O3 group forms both donor and acceptor interactions, the former to a symmetry related carbonyl-O4 and the latter with a symmetry related amine-N1—H atom, Table 1. These lead to a linear supramolecular chain, Fig. 2, aligned along the *a* axis and mediated by an 11-membered {···OH···OCOH···OC_2_NH} synthon; the chain is further stabilized by a C7—H7···O1 contact, Table 1. Chains are held in the crystal structure by C—H···O contacts, Fig. 3 and Table 1.

### Experimental

To 2-amino-3-methylbutanoic acid (234 mg, 2 mmol) in distilled water (15 ml), was added 2,5-dichlorobenzenesulfonyl chloride (491 mg, 2 mmol) while maintaining the pH of reaction mixture at 8 by using 3% sodium carbonate solution. The consumption of the reactants was confirmed by TLC. The pH of reaction mixture was adjusted to 3 using 3 N HCl. The precipitates were washed with water and crystallized from methanol

### Refinement

The C-bound H atoms were geometrically placed (C–H = 0.93–0.98 Å) and refined as riding with *U_iso_*(H) = 1.2–1.5*U_eq_*(C). The O– and N-bound H atoms were refined with the distance restraints O—H = 0.82±0.01 Å and N–H = 0.86±0.01 Å, and with *U_iso_*(H) = *yU_eq_*(parent atom) for *y* = 1.5 (parent atom = O) and *y* = 1.2 (N). In the final refinement four low angle reflections evidently effected by the beam stop were omitted, *i.e*. (002), (012), (011) and (021).

### Crystal data

**Table:**

C_11_H_13_Cl_2_NO_4_S	*F*(000) = 672
*M**_r_* = 326.18	*D*_x_ = 1.451 Mg m^−^^3^
Orthorhombic, *P*2_1_2_1_2_1_	Mo *K*α radiation, λ = 0.71073 Å
Hall symbol: P 2ac 2ab	Cell parameters from 4852 reflections
*a* = 5.4584 (2) Å	θ = 2.6–25.1°
*b* = 14.0623 (6) Å	µ = 0.58 mm^−^^1^
*c* = 19.4545 (8) Å	*T* = 293 K
*V* = 1493.28 (10) Å^3^	Prism, colourless
*Z* = 4	0.19 × 0.13 × 0.07 mm

### Data collection

**Table:**

Bruker APEXII CCD diffractometer	3405 independent reflections
Radiation source: fine-focus sealed tube	2876 reflections with *I* > 2σ(*I*)
graphite	*R*_int_ = 0.040
φ and ω scans	θ_max_ = 27.5°, θ_min_ = 3.5°
Absorption correction: multi-scan (*SADABS*; Sheldrick, 1996)	*h* = −5→7
*T*_min_ = 0.805, *T*_max_ = 0.921	*k* = −18→18
14542 measured reflections	*l* = −25→25

### Refinement

**Table:**

Refinement on *F*^2^	Secondary atom site location: difference Fourier map
Least-squares matrix: full	Hydrogen site location: inferred from neighbouring sites
*R*[*F*^2^ > 2σ(*F*^2^)] = 0.041	H atoms treated by a mixture of independent and constrained refinement
*wR*(*F*^2^) = 0.127	*w* = 1/[σ^2^(*F*_o_^2^) + (0.0887*P*)^2^] where *P* = (*F*_o_^2^ + 2*F*_c_^2^)/3
*S* = 1.00	(Δ/σ)_max_ = 0.001
3405 reflections	Δρ_max_ = 0.61 e Å^−^^3^
180 parameters	Δρ_min_ = −0.51 e Å^−^^3^
2 restraints	Absolute structure: Flack (1983), 1415 Friedel pairs
Primary atom site location: structure-invariant direct methods	Flack parameter: 0.09 (8)

### Special details

**Table:**

Geometry. All s.u.'s (except the s.u. in the dihedral angle between two l.s. planes) are estimated using the full covariance matrix. The cell s.u.'s are taken into account individually in the estimation of s.u.'s in distances, angles and torsion angles; correlations between s.u.'s in cell parameters are only used when they are defined by crystal symmetry. An approximate (isotropic) treatment of cell s.u.'s is used for estimating s.u.'s involving l.s. planes.
Refinement. Refinement of *F*^2^ against ALL reflections. The weighted *R*-factor *wR* and goodness of fit *S* are based on *F*^2^, conventional *R*-factors *R* are based on *F*, with *F* set to zero for negative *F*^2^. The threshold expression of *F*^2^ > 2σ(*F*^2^) is used only for calculating *R*-factors(gt) *etc*. and is not relevant to the choice of reflections for refinement. *R*-factors based on *F*^2^ are statistically about twice as large as those based on *F*, and *R*- factors based on ALL data will be even larger.

### Fractional atomic coordinates and isotropic or equivalent isotropic displacement parameters (Å^2^)

**Table:**

	*x*	*y*	*z*	*U*_iso_*/*U*_eq_	
Cl1	0.36922 (17)	0.23427 (6)	0.14486 (5)	0.0617 (3)	
Cl2	1.2300 (2)	0.15728 (10)	0.34471 (6)	0.0952 (4)	
S1	0.59333 (11)	0.01761 (4)	0.15989 (3)	0.03515 (17)	
O1	0.3334 (3)	0.01414 (15)	0.16803 (12)	0.0491 (5)	
O2	0.7383 (4)	−0.06093 (14)	0.18173 (11)	0.0485 (5)	
O3	1.1937 (3)	0.12919 (13)	0.00395 (12)	0.0465 (5)	
H3O	1.231 (8)	0.1812 (14)	−0.0118 (19)	0.070*	
O4	0.8427 (4)	0.19737 (14)	0.03380 (13)	0.0554 (6)	
N1	0.6435 (4)	0.03271 (15)	0.07885 (11)	0.0359 (5)	
H1N	0.528 (4)	0.0668 (18)	0.0625 (15)	0.043*	
C1	0.7060 (5)	0.11804 (19)	0.20583 (13)	0.0375 (6)	
C2	0.6085 (6)	0.2087 (2)	0.19980 (15)	0.0475 (7)	
C3	0.6997 (8)	0.2826 (2)	0.23875 (19)	0.0671 (10)	
H3	0.6316	0.3430	0.2348	0.080*	
C4	0.8904 (8)	0.2675 (3)	0.28335 (19)	0.0740 (12)	
H4	0.9526	0.3172	0.3096	0.089*	
C5	0.9879 (7)	0.1778 (3)	0.28870 (16)	0.0615 (10)	
C6	0.8996 (5)	0.1019 (2)	0.25120 (14)	0.0453 (6)	
H6	0.9673	0.0416	0.2560	0.054*	
C7	0.8902 (5)	0.02917 (16)	0.05012 (13)	0.0336 (5)	
H7	1.0025	0.0063	0.0858	0.040*	
C8	0.9042 (6)	−0.0395 (2)	−0.01197 (18)	0.0525 (8)	
H8	1.0741	−0.0392	−0.0283	0.063*	
C9	0.7472 (9)	−0.0072 (3)	−0.0703 (2)	0.0855 (13)	
H9A	0.7767	−0.0468	−0.1097	0.128*	
H9B	0.7860	0.0576	−0.0815	0.128*	
H9C	0.5778	−0.0117	−0.0574	0.128*	
C10	0.8466 (11)	−0.1396 (2)	0.0105 (3)	0.103 (2)	
H10A	0.6806	−0.1426	0.0266	0.155*	
H10B	0.9559	−0.1578	0.0468	0.155*	
H10C	0.8666	−0.1821	−0.0278	0.155*	
C11	0.9694 (5)	0.12818 (16)	0.02879 (14)	0.0352 (6)	

### Atomic displacement parameters (Å^2^)

**Table:**

	*U*^11^	*U*^22^	*U*^33^	*U*^12^	*U*^13^	*U*^23^
Cl1	0.0687 (5)	0.0524 (4)	0.0638 (5)	0.0192 (4)	0.0005 (4)	0.0038 (4)
Cl2	0.0659 (6)	0.1600 (11)	0.0597 (6)	−0.0443 (7)	−0.0190 (5)	0.0070 (7)
S1	0.0295 (3)	0.0337 (3)	0.0422 (3)	−0.0026 (2)	0.0021 (3)	0.0031 (3)
O1	0.0314 (9)	0.0567 (11)	0.0593 (13)	−0.0080 (9)	0.0077 (9)	−0.0015 (10)
O2	0.0518 (11)	0.0383 (10)	0.0553 (12)	0.0021 (9)	−0.0007 (10)	0.0110 (8)
O3	0.0367 (10)	0.0367 (10)	0.0661 (13)	−0.0051 (8)	0.0044 (10)	0.0114 (9)
O4	0.0589 (13)	0.0357 (9)	0.0717 (15)	0.0117 (9)	0.0111 (11)	0.0116 (10)
N1	0.0282 (11)	0.0421 (11)	0.0375 (12)	0.0024 (9)	−0.0012 (9)	0.0013 (9)
C1	0.0346 (13)	0.0432 (14)	0.0347 (13)	−0.0069 (11)	0.0054 (11)	−0.0010 (11)
C2	0.0564 (19)	0.0437 (14)	0.0423 (16)	−0.0038 (15)	0.0099 (14)	−0.0035 (11)
C3	0.097 (3)	0.0490 (17)	0.055 (2)	−0.0171 (19)	0.011 (2)	−0.0090 (15)
C4	0.093 (3)	0.074 (2)	0.055 (2)	−0.042 (2)	0.002 (2)	−0.0117 (18)
C5	0.0509 (19)	0.094 (3)	0.0393 (16)	−0.0319 (18)	0.0020 (15)	−0.0026 (17)
C6	0.0367 (14)	0.0618 (16)	0.0375 (14)	−0.0073 (14)	0.0050 (12)	0.0050 (12)
C7	0.0283 (12)	0.0323 (11)	0.0402 (13)	0.0020 (10)	−0.0001 (10)	0.0013 (10)
C8	0.0497 (17)	0.0456 (15)	0.0623 (19)	−0.0054 (14)	0.0187 (16)	−0.0146 (13)
C9	0.086 (3)	0.115 (3)	0.056 (2)	−0.018 (3)	−0.005 (2)	−0.036 (2)
C10	0.150 (5)	0.0403 (17)	0.119 (4)	−0.019 (2)	0.050 (4)	−0.026 (2)
C11	0.0336 (13)	0.0321 (12)	0.0399 (13)	0.0004 (10)	−0.0042 (11)	0.0024 (10)

### Geometric parameters (Å, °)

**Table:**

Cl1—C2	1.725 (3)	C4—C5	1.372 (6)
Cl2—C5	1.737 (4)	C4—H4	0.9300
S1—O2	1.424 (2)	C5—C6	1.380 (5)
S1—O1	1.4286 (19)	C6—H6	0.9300
S1—N1	1.614 (2)	C7—C11	1.516 (3)
S1—C1	1.781 (3)	C7—C8	1.549 (4)
O3—C11	1.317 (3)	C7—H7	0.9800
O3—H3o	0.82 (3)	C8—C9	1.494 (6)
O4—C11	1.197 (3)	C8—C10	1.506 (5)
N1—C7	1.459 (3)	C8—H8	0.9800
N1—H1n	0.853 (10)	C9—H9A	0.9600
C1—C2	1.387 (4)	C9—H9B	0.9600
C1—C6	1.395 (4)	C9—H9C	0.9600
C2—C3	1.379 (5)	C10—H10A	0.9600
C3—C4	1.372 (6)	C10—H10B	0.9600
C3—H3	0.9300	C10—H10C	0.9600
			
O2—S1—O1	119.51 (12)	N1—C7—C11	109.66 (19)
O2—S1—N1	107.41 (12)	N1—C7—C8	111.5 (2)
O1—S1—N1	106.34 (13)	C11—C7—C8	110.2 (2)
O2—S1—C1	105.87 (13)	N1—C7—H7	108.5
O1—S1—C1	108.33 (13)	C11—C7—H7	108.5
N1—S1—C1	109.10 (12)	C8—C7—H7	108.5
C11—O3—H3O	112 (3)	C9—C8—C10	112.6 (4)
C7—N1—S1	121.76 (17)	C9—C8—C7	112.0 (3)
C7—N1—H1N	124 (2)	C10—C8—C7	110.3 (3)
S1—N1—H1N	108 (2)	C9—C8—H8	107.2
C2—C1—C6	119.6 (3)	C10—C8—H8	107.2
C2—C1—S1	123.7 (2)	C7—C8—H8	107.2
C6—C1—S1	116.8 (2)	C8—C9—H9A	109.5
C3—C2—C1	120.5 (3)	C8—C9—H9B	109.5
C3—C2—Cl1	117.2 (3)	H9A—C9—H9B	109.5
C1—C2—Cl1	122.3 (2)	C8—C9—H9C	109.5
C4—C3—C2	120.3 (4)	H9A—C9—H9C	109.5
C4—C3—H3	119.8	H9B—C9—H9C	109.5
C2—C3—H3	119.8	C8—C10—H10A	109.5
C3—C4—C5	119.0 (3)	C8—C10—H10B	109.5
C3—C4—H4	120.5	H10A—C10—H10B	109.5
C5—C4—H4	120.5	C8—C10—H10C	109.5
C6—C5—C4	122.3 (3)	H10A—C10—H10C	109.5
C6—C5—Cl2	118.0 (3)	H10B—C10—H10C	109.5
C4—C5—Cl2	119.7 (3)	O4—C11—O3	123.9 (2)
C5—C6—C1	118.3 (3)	O4—C11—C7	124.0 (2)
C5—C6—H6	120.9	O3—C11—C7	112.0 (2)
C1—C6—H6	120.9		
			
O2—S1—N1—C7	−44.1 (2)	C3—C4—C5—C6	−0.6 (6)
O1—S1—N1—C7	−173.12 (18)	C3—C4—C5—Cl2	179.8 (3)
C1—S1—N1—C7	70.2 (2)	C4—C5—C6—C1	0.8 (5)
O2—S1—C1—C2	−178.7 (2)	Cl2—C5—C6—C1	−179.6 (2)
O1—S1—C1—C2	−49.4 (3)	C2—C1—C6—C5	−0.1 (4)
N1—S1—C1—C2	66.0 (3)	S1—C1—C6—C5	−178.7 (2)
O2—S1—C1—C6	−0.2 (2)	S1—N1—C7—C11	−108.9 (2)
O1—S1—C1—C6	129.1 (2)	S1—N1—C7—C8	128.8 (2)
N1—S1—C1—C6	−115.5 (2)	N1—C7—C8—C9	63.4 (3)
C6—C1—C2—C3	−0.7 (4)	C11—C7—C8—C9	−58.6 (3)
S1—C1—C2—C3	177.8 (3)	N1—C7—C8—C10	−62.8 (4)
C6—C1—C2—Cl1	−180.0 (2)	C11—C7—C8—C10	175.2 (3)
S1—C1—C2—Cl1	−1.6 (4)	N1—C7—C11—O4	−1.6 (4)
C1—C2—C3—C4	0.8 (5)	C8—C7—C11—O4	121.4 (3)
Cl1—C2—C3—C4	−179.8 (3)	N1—C7—C11—O3	178.5 (2)
C2—C3—C4—C5	−0.2 (6)	C8—C7—C11—O3	−58.4 (3)

### Hydrogen-bond geometry (Å, °)

**Table:**

*D*—H···*A*	*D*—H	H···*A*	*D*···*A*	*D*—H···*A*
O3—H3o···O4^i^	0.82 (3)	1.86 (2)	2.674 (3)	171 (3)
N1—H1n···O3^ii^	0.85 (2)	2.32 (2)	3.161 (3)	167 (3)
C7—H7···O1^iii^	0.98	2.42	3.341 (3)	157
C4—H4···O2^iv^	0.93	2.41	3.223 (5)	146

Symmetry codes: (i) *x*+1/2, −*y*+1/2, −*z*; (ii) *x*−1, *y*, *z*; (iii) *x*+1, *y*, *z*; (iv) −*x*+2, *y*+1/2, −*z*+1/2.
